# Asoprisnil as a Novel Ligand Interacting with Stress-Associated Glucocorticoid Receptor

**DOI:** 10.3390/biomedicines12122745

**Published:** 2024-11-30

**Authors:** Ovinuchi Ejiohuo, Donald Bajia, Joanna Pawlak, Aleksandra Szczepankiewicz

**Affiliations:** 1 Department of Psychiatric Genetics, Poznan University of Medical Sciences, 61-701 Poznan, Poland; jopawlak@ump.edu.pl; 2 Doctoral School, Poznan University of Medical Sciences, Bukowska 70, 60-812 Poznan, Poland; bajiadonald8@gmail.com; 3 Molecular and Cell Biology Unit, Poznan University of Medical Sciences, 60-572 Poznan, Poland; alszczep@ump.edu.pl; 4 Department of Pediatric Oncology, Hematology and Transplantology, Poznan University of Medical Sciences, 60-572 Poznan, Poland

**Keywords:** glucocorticoid receptor, asoprisnil, in silico drug target profiling, ligand–protein interaction, computational chemistry, stress-related mental disorders, targeted therapy, hypothalamic–pituitary–adrenal (HPA) axis, molecular docking, molecular dynamic simulation

## Abstract

**Background/objective:** The glucocorticoid receptor (GR) is critical in regulating cortisol production during stress. This makes it a key target for treating conditions associated with hypothalamic–pituitary–adrenal (HPA) axis dysregulation, such as mental disorders. This study explores novel ligands beyond mifepristone for their potential to modulate GR with improved efficacy and safety. By investigating these interactions, we seek to identify new pharmacotherapeutic options for stress-related mental illness. **Methods:** The ligands asoprisnil, campestanol, and stellasterol were selected based on structural similarities to mifepristone (reference ligand) and evaluated for pharmacological and ADME (absorption, distribution, metabolism, and excretion) properties using the SwissADME database. Molecular docking with AutoDock 4.2.6 and molecular dynamics simulations were performed to investigate ligand–protein interactions with the human glucocorticoid receptor, and binding free energies were calculated using MMPBSA. **Results:** Pharmacokinetic analysis revealed that asoprisnil exhibited high gastrointestinal absorption and obeyed Lipinski’s rule, while mifepristone crossed the blood–brain barrier. Toxicological predictions showed that mifepristone was active for neurotoxicity and immunotoxicity, while asoprisnil, campestanol, and stellasterol displayed lower toxicity profiles. Asoprisnil demonstrated the highest stability in molecular dynamics simulations, with the highest negative binding energy of −62.35 kcal/mol, when compared to mifepristone, campestanol, and stellasterol, with binding energies of −57.08 kcal/mol, −49.99 kcal/mol, and −46.69 kcal/mol, respectively. **Conclusion:** This makes asoprisnil a potentially favourable therapeutic candidate compared to mifepristone. However, further validation of asoprisnil’s interaction, efficacy, and safety in stress-related mental disorders through experimental studies and clinical trials is needed.

## 1. Introduction

Cortisol is a steroid hormone (also known as a stress hormone) and the main glucocorticoid produced by the adrenal glands from cholesterol in response to stress [1,2]. Stress can serve as a trigger or distal factor, modulating susceptibility to mental disorders over time. Stress has been connected to physiological dysregulations that can exacerbate or alter clinical conditions, impacting stress responses, which is critical to understanding and managing such conditions. In the study by Krahel et al. (2021), stress exacerbated attention-deficit/hyperactivity disorder (ADHD) symptoms by contributing to HPA axis hyperactivity and dysregulation, increasing immune markers (sAA, IgA, IgM), and affecting metabolic health, as seen in higher body mass and BMI; this interplay suggests that stress may heighten ADHD symptoms through hormonal, immune, and metabolic pathways, ultimately increasing broader health risks [3]. The hypothalamic–pituitary–adrenal (HPA) axis regulates cortisol secretion in response to stress, and its dysregulation was previously observed in bipolar patients, thus highlighting the role of cortisol in stress-associated bipolar disorder [4]. However, the pathophysiological mechanisms of the dysregulated HPA axis remain unclear [5]. There is a complex interaction between stress, cortisol regulation, and bipolar disorder, with individual variability. The study by Manenschijn et al. (2012) found significantly lower hair cortisol levels in bipolar disorder patients with panic disorder and higher saliva cortisol in bipolar disorder patients with panic disorder [6]. In another study by Faresjo et al. (2013), young Greek adults with higher stress levels and stressful life events had significantly lower cortisol levels when compared to young Swedish adults [7]. In a more recent study by Lyu et al. (2023), lower adrenocorticotrophic hormone (ACTH) was observed in bipolar disorder patients with mania, thus resulting in low cortisol levels since ACTH controls cortisol production [8,9]. Cortisol levels are usually high in patients with mental illness due to chronic stress [10]. Psychiatric disorders are linked to high cortisol levels [11], encompassing mental health conditions, including anxiety, depression, mania, and hypomania, and other mental health symptoms like mood, sleep, and energy disturbances [12].

The glucocorticoid receptor (GR) regulates cortisol production through a negative feedback loop in the hypothalamic–pituitary–adrenal (HPA) axis: cortisol release is stimulated by stress, but as cortisol levels rise, they activate GRs in the hypothalamus and pituitary, inhibiting the further release of corticotropin-releasing hormone (CRH) and adrenocorticotropic hormone (ACTH) to maintain homeostasis [2,13,14,15,16]. Understanding the role of GR in regulating cortisol production and targeting GR represents a promising approach to developing novel, safe, and effective therapeutics that selectively target GR with minimal side effects for conditions associated with dysregulation of the HPA axis, including bipolar disorder. The strategy of targeting GR with drugs that target HPA abnormalities for mental disorders shows that GR has one of the most promising results [17].

The *NR3C1* (Nuclear Receptor Subfamily 3, Group C, Member 1) gene produces two main isoforms (GR-α and GR-β) of the glucocorticoid receptor (GR) through alternative splicing [18,19]. GR-α is the active isoform, mediating biological effects [19,20] such as maintaining homeostasis, regulating inflammation, and responding to stress [21]. GR-β acts as a dominant negative inhibitor, blocking the action of ligand-activated GR-α. The production of these isoforms varies based on neuronal conditions, influencing their responsiveness to cortisol [22]. Understanding these isoforms is crucial as their balance and expression levels affect glucocorticoid sensitivity and cellular responses to stress and inflammation. The effects of strong interacting ligands such as mifepristone on glucocorticoid receptor (GR) can impact various physiological processes, including stress response regulation in mental illness. Mifepristone functions as a GR antagonist, impeding the receptor’s release from related heat shock proteins and thus blocking the receptor’s translocation into the nucleus [23]. This blocks the cortisol effect since mifepristone interferes with cortisol binding to the receptor. In this case, it decreases the effects of excess cortisol, such as elevated blood sugar levels, rather than decreasing cortisol production [24]. The study by Blasey et al. (2011) assessed the safety and effectiveness of mifepristone for treating psychotic depression, with a secondary goal of determining if patients with higher mifepristone plasma levels showed greater improvement [25]. While this study did not find a significant difference in response rates between mifepristone and placebo overall, it did show that patients with mifepristone plasma concentrations above 1660 ng/mL had a significantly better reduction in psychotic symptoms, suggesting that mifepristone might be effective at higher plasma levels, highlighting the need for further research. As an antagonist, it can result in either normal or elevated cortisol levels [26]. In the former, mifepristone reduces cortisol clearance and furthers cortisol production [27] by blocking GR. In the study by Garner et al. (2016), patients suffering from psychosis, when treated with mifepristone, had their cortisol levels normalised [28]. Mifepristone also facilitates cortisol penetration through the blood–brain barrier, resetting the stress system [27].

While mifepristone is effective for some, its efficacy varies among individuals [29]. The exact percentage of patients resistant to mifepristone is not explicit, and this is for its gynaecological application. Studies affirm high overall effectiveness, with success rates approaching 99.6% in certain protocols [30,31]. Consequently, research into novel ligands may lead to molecules with improved efficacy and safety profiles and offer other therapeutic options. Hence, research is also needed to elucidate the interaction of GR with potential molecules and to study the structural changes induced by their binding that result in receptor antagonism since mifepristone shows direct interaction with GR [32]. Therefore, this study aims to investigate the interaction of alternative ligands with glucocorticoid receptor (GR) beyond mifepristone and to analyse how these interactions potentially influence various physiological processes, particularly stress response regulation in mental illness. Absorption, distribution, metabolism, and excretion (ADME) and toxicity analyses were used to determine if the pharmacokinetic (PK) and pharmacodynamic (PD) properties of our identified ligands fall within an acceptable range [33] as this is necessary for drug discovery and development. Then, using in silico methods such as molecular docking and molecular dynamic simulation, we identified the most promising ligand candidate that interacted with GR, and we investigated the structural changes induced by the ligand binding that might result in receptor modulation.

## 2. Materials and Methods

### 2.1. Ligand Selection

The Simplified Molecular Input Line Entry System (SMILE) of mifepristone, asoprisnil, campestanol, and stellasterol was obtained from PubChem (https://pubchem.ncbi.nlm.nih.gov/ (21 February 2024)). These ligands were selected based on their structural similarities.

### 2.2. Ligand Properties Screening

The ligands’ pharmacological and ADME (absorption, distribution, metabolism, and excretion) properties were accessed using SwissADME [34]. ProTox 3.0 and STopTox were used to predict the toxicity of the ligands by using the SMILEs of the ligands. ProTox 3.0 predicts the toxicity of small molecules by combining molecular similarity, fragment propensities, frequent structural features, and machine learning techniques based on 61 models, covering endpoints like acute and organ toxicity, molecular initiating events, metabolism, and Tox21 pathways [35]. STopTox predicts a molecule’s toxicity by using externally validated quantitative structure–activity relationship (QSAR) models to rapidly assess each of the “6-pack” acute toxicity endpoints: oral, dermal, and inhalation toxicity, as well as skin and eye irritation and skin sensitisation, based on experimental animal data [36].

### 2.3. Protein Preparation and Quality Check

The protein data bank (PDB ID: 1NHZ) provided the human glucocorticoid receptor protein’s 3D structure in PDB format. The Molegro Molecular Viewer was used to remove the Hexane-1,6-Diol bonded to the protein (http://molexus.io/molegro-molecular-viewer/ (21 February 2024)). PrankWeb [37,38,39] was used to determine the protein’s binding site and the binding site residues (A_560 A_563 A_564 A_567 A_568 A_570 A_571 A_600 A_601 A_604 A_605 A_608 A_611 A_623 A_639 A_642 A_643 A_646 A_732 A_735 A_736 A_737). ERRAT on the SAVESv6.0 (structural validation server) was used to verify the protein structure quality [40].

### 2.4. Molecular Docking

AutoDock 4.2.6 was used for the molecular docking to investigate the ligand–protein interaction [41]. Polar hydrogens and Kollman charges were added to the protein, and water molecules and heteroatoms were eliminated in preparation for the docking process. The ligand molecule had charges and hydrogens added to it. A maximum of 32 torsion bonds were checked and permitted for the ligands. Coordinates acquired from PrankWeb were used to determine the protein’s active site and to adjust the Grid box axis parameters. The grid box X, Y, and Z centre coordinates (−1.9294, 12.9952, and 4.2211, respectively) were from the predicted protein binding pocket data from the PrankWeb binding site prediction server. To ensure consistency across simulations, docking parameters, including the genetic algorithm, were set to 50 runs and a population size of 300 for all ligand–protein complexes.

### 2.5. Molecular Docking Analysis

Several servers were used for the post-docking analysis. The 3D ligand–protein complex was visualised, and the interaction surfaces were created using NCBI’s iCn3D Structure Viewer (https://www.ncbi.nlm.nih.gov/Structure/icn3d/ (5 October 2024)) [42,43]. Protein Plus (https://proteins.plus/ (12 October 2024)) pose view was utilised to show the 2D interaction diagrams of the complex’s binding poses [44]. The interaction was further confirmed using a 2D interaction diagram from LigPlot Plus [45].

### 2.6. Molecular Dynamic Simulation

Molecular dynamic simulation was conducted using SiBioLead (https://sibiolead.com (2 March 2024 to 12 March 2024)). Molecular mechanics Poisson–Boltzmann surface area (MMPBSA) was used to calculate the ligand’s binding free energy. The complex was inserted into a triclinic box filled with 0.15 M NaCl-containing simple point charge (SPC) water, and the optimised potentials for liquid simulations all-atoms (OPLS/AA) forcefield was used. The steepest descent integrator was used for 5000 steps to minimise the system’s energy consumption. For 100 ps, equilibration was conducted at 300 K in temperature and 1 bar in pressure. With 5000 frames saved and a simulation time of 100 ns, the leap frog integrators were utilised as the simulation parameter. The simulation results were plotted using QtGrace Version 0.2.7 software and then analysed. Output data files in xvg format of each complex from the MD simulation for each property—root mean square deviation (RMSD), root mean square fluctuation (RMSF), radius of gyration, solvent-accessible surface area (SASA), and hydrogen bonds—were used to create the plots. The plots were then analysed to interpret the stability, flexibility, and interactions observed in the MD simulation.

## 3. Results and Discussion

### 3.1. Pharmacokinetic and Toxicological Analysis of the Ligands

SwissADME was used to predict our ligands’ pharmacokinetic properties. According to the BOILED-Egg model in Figure S1 (Supplementary Materials), mifepristone has a high gastrointestinal (GI) absorption and can also cross the blood–brain barrier. This is followed only by asoprisnil, which only has high GI absorption. Campestanol and stellastanol have no potential for GI absorption and crossing the blood–brain barrier (BBB). Asoprisnil is the only ligand (highlighted in blue) substrate for P-glycoprotein (PGP+). P-glycoprotein, or ABCB1, is a membrane-bound protein encoded by the *ABCB1* gene in humans that protects the body by reducing drug absorption in the intestines and facilitating drug excretion from cells through the kidneys and liver into the urine and bile [46,47]. This protects the body from potentially harmful drug accumulation and maintains physiological homeostasis.

Table 1A shows the pharmacokinetic properties of the ligands. Only asoprisnil obeyed Lipinki’s rule (molecular weight: < 500; MlogP: ≤ 4.15; number of hydrogen-bond acceptors: < 10; number of hydrogen-bond donors: < 5) [48]. The consensus Log P_o/w_ value refers to the average value of a molecule’s lipophilicity (Log P) predicted by five different methods from the SwissADME web tool [34], which offers a more accurate measure of a molecule’s lipophilicity than relying on a single prediction method.

The results of the toxicity profile of our ligands are presented in Table 1B. All four ligands are predicted to have a toxicity class of 4, with mifepristone having an oral LD50 of 680 mg/kg and prediction accuracy of 67.38%, asoprisnil having an LD50 of 1520 mg/kg and an accuracy of 67.38%, campestanol having an LD50 of 500 mg/kg and an accuracy of 100%, and stellasterol having an LD50 of 2000 mg/kg and an accuracy of 100%.

The result from the ToxPro 3.0 server (Table 1B) for our reference ligand, mifepristone, indicate that it was active for neurotoxicity, immunotoxicity, and toxicity for stress response pathways for mitochondrial membrane potential (MMP) and phosphoprotein (tumour suppressor) p53. Asoprisnil and campestanol were only active for immunotoxicity, and stellasterol was active only for immunotoxicity and partially for neurotoxicity. Besides the reference ligands, all are predicted to be non-toxic to stress response pathways. This implies that they can safely support cellular survival under adverse conditions without disrupting essential protective mechanisms [49,50], enhancing its therapeutic safety profile. Asoprisnil was inactive for any Cytochrome P450 (CYP) enzymes. Mifepristone was active for CYP2C19, CYP2D6, and CYP3A4. Studies recommend a controlled dose and the avoidance of concurrent use of mifepristone as an inhibitor and inducer of CYP34A [51,52,53,54]. Campestanol and stellasterol were active for only CYP2C9. The inhibition or induction of these enzymes can result in increased plasma concentration, altered drug efficacy, or an increased risk of adverse effects form either the ligands or the co-administered drugs, depending on the drug administered or its therapeutic range [55,56,57,58]. This information helps in managing drug interactions and optimising therapeutic outcomes.

The toxicity profile of the ligands does not necessarily invalidate the ligands as potential pharmacotherapeutic agents. Instead, it provides the necessary safety profile to advance the molecules for clinical use. For instance, our reference ligand, mifepristone, from Table 1B, shows active neurotoxicity, immunotoxicity, and toxicity to stress response pathways. However, it has been shown in a human clinical study to alleviate symptoms and improve neurocognitive function and mood in bipolar disorder and psychotic depression [59,60]. Animal studies also show that it might improve anxiety symptoms [61]. The pharmacokinetic and toxicity results favour asoprisnil and make it a preferred pharmacotherapeutic alternative to mifepristone for stress-related mental disorders. However, there are limited or no studies and clinical trials that explore asoprisnil’s role in mental disorders, hence making our study highly relevant as a basis for further investigations. Asoprisnil’s primary therapeutic application is in gynaecological conditions such as uterine fibroid and heavy menstruation [62,63], similar to mifepristone.

In the case of campestanol, there are no direct studies on its role in mental disorders. However, studies suggest it might influence brain health by modulating cholesterol metabolism, a pathway connected with neurological disorders [64,65]. Also, for stellasterol, no evidence shows their application in mental disorders. However, it might be promising due to its neuroprotective and anti-inflammatory properties and might potentially have therapeutic benefits for oxidative stress and neuroinflammation-related mental disorders [66,67].

### 3.2. Protein Preparation and Quality Check Result

**Figure 1:**
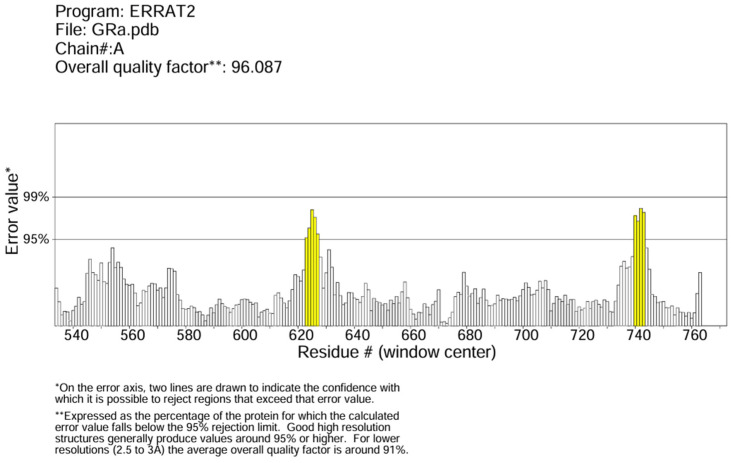
ERRAT plot for GR.

The predicted best binding pocket from PrankWeb scores 21.55 compared to 2.45 and 1.93 for the other predicted pockets. It has 59 surface atoms with x, y, and z coordinates of −1.9294, 12.9952, and 4.2211, respectively, with the binding site residues in Section 2.3. The ERRAT score for GR is about 96% and did not exceed the 99% threshold error value (Figure 1).

### 3.3. AutoDock Result Analysis

The binding affinities for the ligand–protein complex are presented in Table 2. Our reference mifepristone–GR complex had the lowest negative scoring value, indicating a higher binding affinity for the protein. This was followed by the stellasterol–GR complex and the asoprisnil–GR complex. It is, however, essential to acknowledge the limitations of predicting binding affinity using docking. A significant drawback of classical docking is that it offers a static view of the binding conformation, neglecting the dynamic interactions and environmental factors like water, which are more accurately represented through molecular dynamics (MD) simulations that validate the results of classical docking [68].

The inhibition constant (Ki) value from the docking results gives us insight into which ligands have a higher chance of modulating the protein’s function and helps us decide which ligand to investigate further. A lower Ki indicates a tighter binding/interaction between the ligands and the protein [69]. The inhibition constants (Ki) are calculated using the empirical relationship between binding free energy (ΔG) and Ki, following the formula ΔG = RT in Ki, where R is the gas constant (1.987 cal K^−1^ mol^−1^) and T is the temperature in Kelvin (298.15 K) [70]. It is important to consider all results and analysis of the study to decide on the lead molecule. From Table 2, mifepristone has the lowest Ki value of 821.48 pM, followed by stellasterol (1.41 nM) and asoprisnil (2.76 nM).

### 3.4. Molecular Interaction Analysis

**Figure 2:**
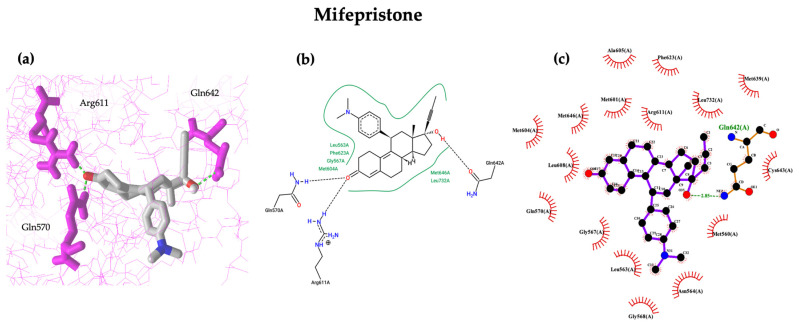

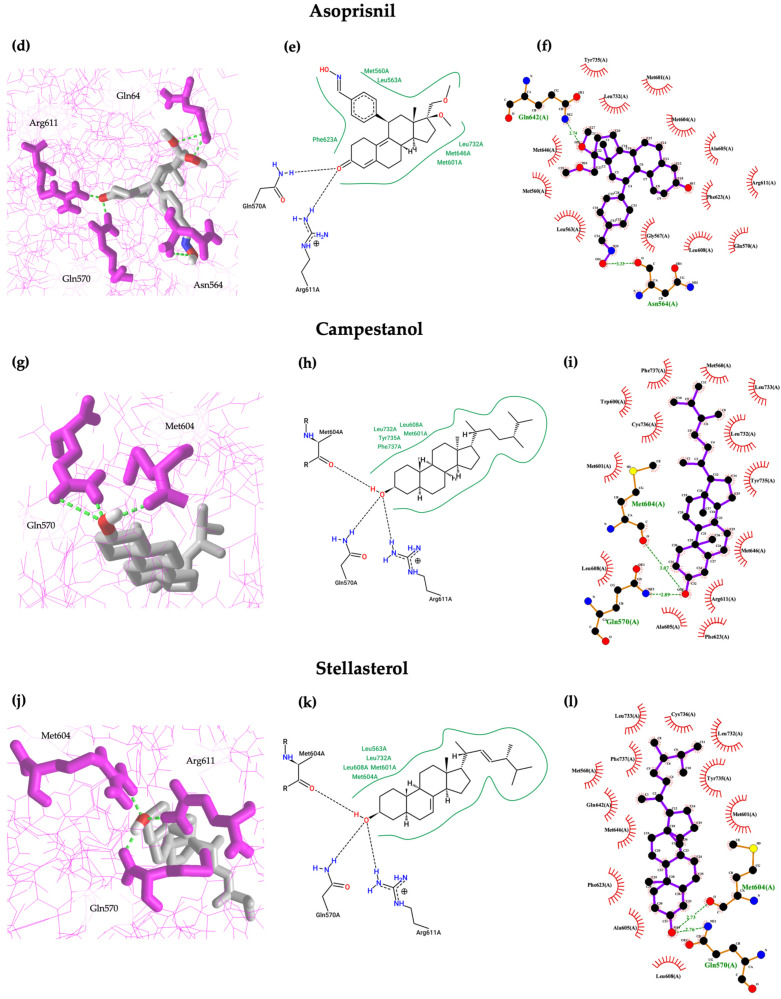
Molecular interaction visualisation of mifepristone-GR complexes (**a**–**c**), asoprisnil-GR complex (**d**–**f**), campestanol-GR complex (**g**–**i**), and stellasterol-GR complex (**j**–**l**). (**a**,**d**,**g**,**j**) are from NCBI’s iCn3D Structure Viewer, (**b**,**e**,**h**,**k**) are from ProteinsPlus pose view, and (**c**,**f**,**i**,**l**) are from LigPlot+. Dashed green and dashed black lines represent hydrogen bonds. Bold green lines and eye-like icons represent hydrophobic interactions.

Visualisation of the interaction of our ligands and proteins was conducted using NCBI’s iCn3D Structure Viewer, ProteinsPlus pose view, and LigPlot+. From Figure 2a,b, mifepristone formed one hydrogen bond each with Arg611, Gln570, and Gln642 making a total of 3 hydrogen bonds formed; only one hydrogen bond with Gln642 in Figure 2c has a bond length of 2.83 Å. For asoprisnil, two hydrogen bonds are observed with Gln642 and one each with Arg611, Gln570, and Asn564 (Figure 2d). From Figure 2e, asoprisnil formed two hydrogen bonds with Gln570 and Arg611. In Figure 2f, we can see that it formed two hydrogen bonds with Gln642, with a bond length of 2.74 Å, and Asn564, with a bond length of 3.33 Å. From Figure 2g, campestanol formed two hydrogen bonds with Gln570 and one hydrogen bond with Met604. In Figure 2h, three hydrogen bonds were formed with Gln570, Met604, and Arg611; while in Figure 2i, it formed two hydrogen bonds with Gln570, with a bond length of 2.89 Å, and Met604, with a bond length of 3.07 Å. In Figure 2j,k, stellasterol formed three hydrogen bonds with Gln570, Arg611, and Met604. In Figure 2l, it formed two hydrogen bonds with Gln570, with a bond length of 2.76 Å, and Met604, with a bond length of 2.73 Å. All complexes interacted with Gln570 and Arg611, thus suggesting these amino acid residues to be potential drug-binding surface hotspots. These ligands, therefore, potentially alter the protein function and point us to key molecules that can modulate the proteins containing them. This can aid in optimising drug candidates by focusing on the regions likely to yield successful binding interactions [71].

Similar interaction patterns can be observed in the mifepristone–GR and asoprisnil–GR complexes. This is also observed for the campestanol–GR complex and the stellasterol–GR complex. These ligands may function similarly when interacting with GR. Figure 2 shows that more hydrogen bonds and interacting amino acid residue are observed for the asoprisnil–GR complex than other complexes, including our reference complex. Hydrogen bonds regulate the specificity of ligand–protein interactions, while hydrophobic interactions enhance the stability of docked complexes [72,73].

### 3.5. Molecular Mechanics Poisson–Boltzmann Surface Area (MMPBSA) Calculations

To confirm the true binding energy (how strong the ligands interact with the GR protein), corroborate the result of the binding affinity from the molecular docking result, and eliminate false positives, MMPBSA free binding energy was calculated. The free binding energy values for mifepristone, asoprisnil, campestanol, and stellasterol are −57.08 kcal/mol, −62.35 kcal/mol, −49.99 kcal/mol, and −46.69 kcal/mol, respectively. The asoprisnil–GR complex scored higher than the reference ligand mifepristone–GR complex, indicating that the asoprisnil–GR complex is the lead candidate and energetically more favourable than our reference complex.

Molecular dynamic simulation was carried out for our lead candidate (asoprisnil–GR complex) to investigate its stability and compare it with our reference complex (mifepristone–GR complex).

### 3.6. Molecular Dynamic Simulation Result

**Figure 3:**
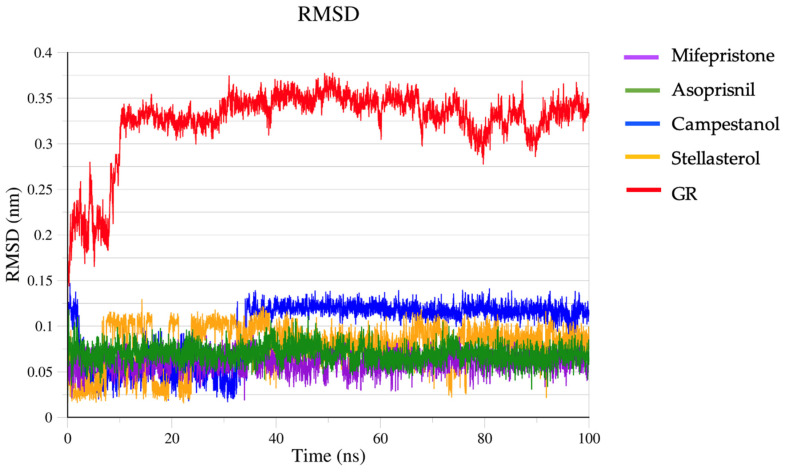
RSMD of the complexes.

Molecular dynamic simulation was used to validate and compare our ligand–protein complexes’ structural stability, fluctuation, and conformational changes. Root mean square deviation (RMSD) gives us information on the stability of our ligand–protein complexes. Stable proteins often have an RSMD below 0.3 nm during simulations [74,75], with possible fluctuations around this range. In Figure 3, the RMSD of GR shows a significant initial increase within the first 10 ns, where it rises to around 0.3 nm, indicating a rapid conformational adjustment as the system equilibrates. After this initial adjustment, it stabilises, oscillating between 0.3 and 0.35 nm for the rest of the simulation. This steady fluctuation suggests that the GR structure reaches equilibrium relatively early and remains stable with minor deviations. Its stable RMSD values beyond the initial 10 ns suggest that GR does not undergo significant conformational changes during the simulation. In MD simulations of ligand–protein complexes, the protein typically shows a higher RMSD than the ligand due to its larger, more flexible structure and the ligand’s restricted movement within a binding pocket. All ligands in the complexes are below 0.15 nm. Mifepristone and asoprisnil remained stable throughout the simulation, with a lower RSMD (0.09 nm) than the other ligands. Campestanol achieved an equilibrium state at about 35 ns through to 100 ns. Stellasterol was fairly stable in a range similar to campestanol.

**Figure 4:**
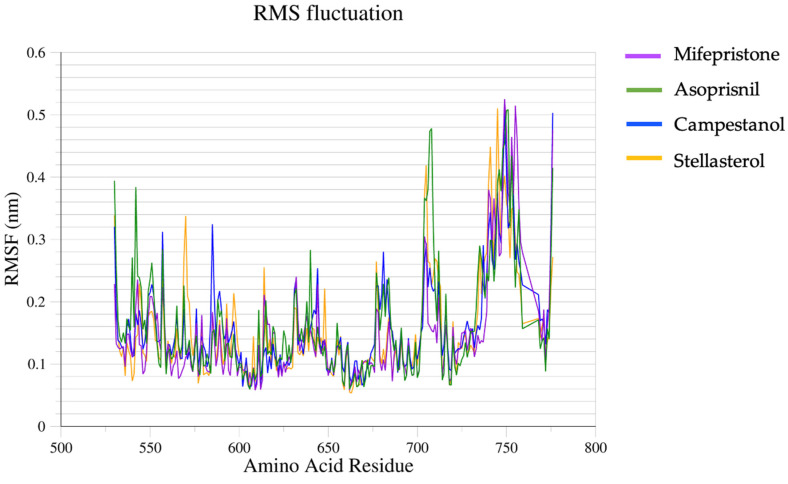
RMSF of the complexes.

The root mean square fluctuation (RMSF) plot depicts the fluctuation of amino acid residues across the four ligand–protein complexes (Figure 4). For residues between approximately 500 and 700, the RMSF values are consistently low across all four complexes, mainly below 0.3 nm. This suggests that these regions are relatively stable with limited flexibility in the protein structure, possibly indicating a core or structured region of the protein. There is a significant increase in RMSF from about 700 to 800, particularly for mifepristone and asoprisnil, which reach RMSF values of up to 0.51 nm. This indicates high flexibility or movement in these regions, potentially pointing to loop regions or parts of the protein that are less structured or more exposed to solvent. While the overall pattern of RMSF values is similar for all, there are noticeable differences in magnitude. Mifepristone exhibits higher fluctuations in certain regions than others, particularly in the high-RMSF zone near residue 750, whereas stellasterol shows a relatively lower RMSF in the same region. Generally, the mifepristone–GR and asoprisnil–GR complexes have the highest stability. In contrast, the stellasterol–GR and campestanol–GR complexes demonstrate more fluctuation, with the campestanol–GR complex being the least stable.

**Figure 5:**
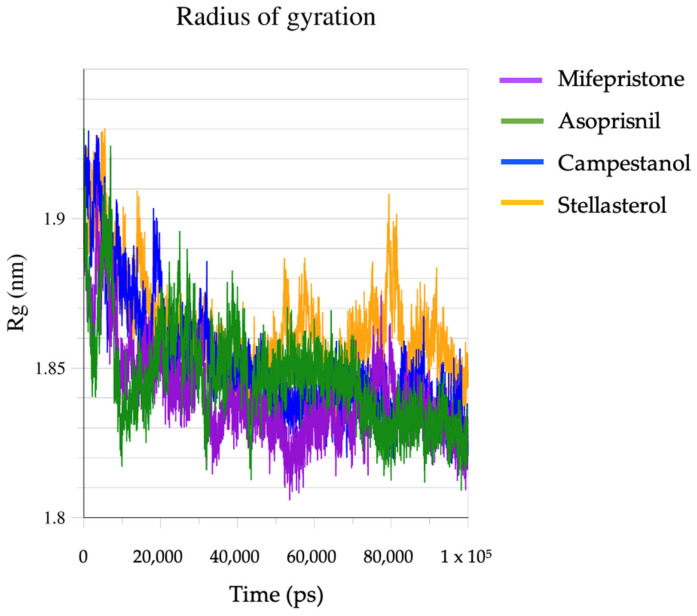
The radius of gyration (Rg) of the complexes.

The radius of gyration (Rg) plot depicts the compactness of the ligand–protein complexes over time (Figure 5). At the start (0–20,000 ps), the Rg values for all complexes are relatively high, fluctuating around 1.85–1.9 nm. This indicates that the protein structure begins in a relatively expanded or less compact state. Across the timeline (up to ~100,000 ps), the Rg values generally decrease and stabilise around 1.82–1.86 nm for most complexes. This suggests that the protein complexes become more compact and stable over time as the system reaches equilibrium. This trend is indicative of structural stabilisation after ligand binding. The mifepristone–GR and asoprisnil–GR complexes show relatively consistent and lower Rg values (~1.83–1.85 nm) throughout most of the simulation, indicating that these complexes maintain a more compact structure. The stellasterol–GR complex exhibits the highest Rg values throughout most of the timeline, fluctuating around 1.85–1.9 nm before stabilising closer to 1.85 nm. This suggests that this complex remains slightly less compact than the others, indicating possible differences in how the ligand interacts with the protein. Campestanol displays a middle-ground behaviour, with Rg values closer to 1.85 nm, showing more variability than the mifepristone and asoprisnil complexes.

**Figure 6:**
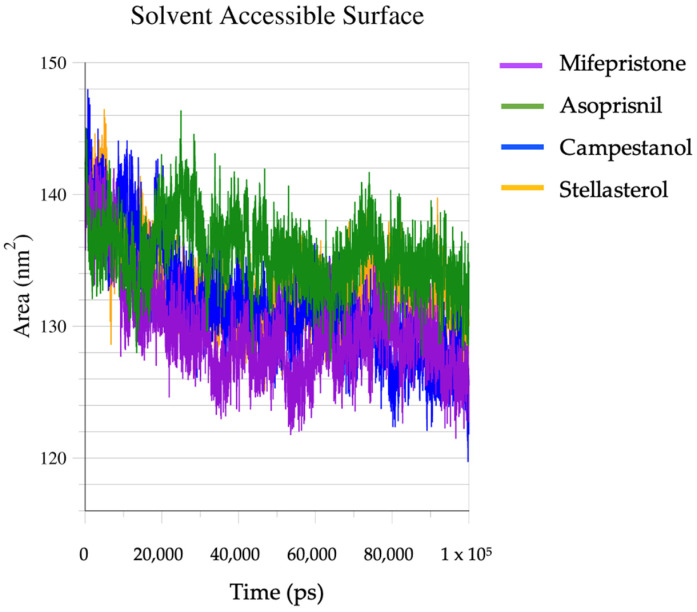
Solvent accessible surface (SASA) of the complexes.

This solvent-accessible surface area (SASA) plot shows how the surface area of the ligand–protein complexes exposed to solvent changes over time (Figure 6). At the beginning of the simulation (0 ps), all four complexes exhibit higher SASA values (~140–150 nm^2^), indicating that more of the protein surface is exposed to the solvent. All complexes show a general downward trend in SASA values, suggesting that the proteins become less solvent-accessible as the simulation progresses. This could be due to the proteins adopting a more compact conformation or ligand-induced stabilisation. This decrease in SASA indicates that the proteins are folding or adjusting to reduce their exposed surface area, which is typical when a ligand binds and stabilises a protein structure. The asoprisnil–GR complex shows the highest SASA values throughout the simulation, indicating that it remains the most solvent-exposed. It fluctuates around 130–140 nm^2^ for most of the timeline, with the least drop compared to the other complexes. The mifepristone–GR and campestanol–GR complexes demonstrate lower SASA values throughout, suggesting they adopt more compact conformations as the simulation progresses. They stabilise around 125–130 nm^2^, indicating reduced solvent exposure. The stellasterol–GR complex shows significant fluctuations early on but follows a trend similar to the asoprisnil–GR complex, stabilising closer to 135–140 nm^2^. Its higher SASA values suggest it remains more open to the solvent than the mifepristone–GR and campestanol–GR complexes. At the end of the simulation (~100,000 ps), the SASA values stabilise across all complexes. The mifepristone–GR and campestanol–GR complexes show the lowest solvent-exposed areas (~125–130 nm^2^), while the asoprisnil–GR and stellasterol–GR complexes exhibit slightly higher SASA values (~135–140 nm^2^).

**Figure 7:**
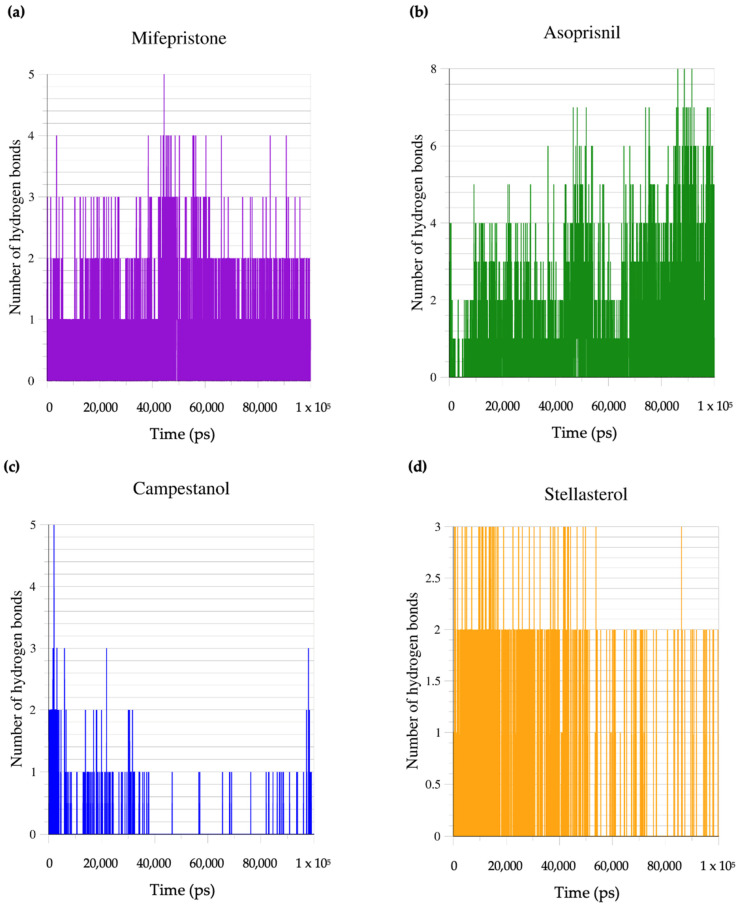
The number of hydrogen bonds formed (**a**) mifepristone-GR complex (**b**) asoprisnil-GR complex (**c**) Campestanol-GR complex (**d**) stellasterol-GR complex.

Regarding the number of hydrogen bonds (Figure 7a–d), the asoprisnil–GR complex showed the highest number of hydrogen bonds, reaching up to eight at certain points during the simulation. The high number of hydrogen bonds indicates a strong interaction between asoprisnil and the GR, suggesting a potentially high binding affinity and stable interaction. Hydrogen bonds play a crucial role in stabilising ligand–protein interactions, and having up to eight bonds suggests that this complex might be the most stable.

The mifepristone–GR complex forms around four hydrogen bonds, which is lower than the asoprisnil–GR complex but still indicates a relatively strong binding interaction. The campestanol–GR and stellasterol–GR complexes show around three hydrogen bonds, suggesting weaker interactions with the GR protein than the asoprisnil–GR and mifepristone–GR complexes. The fewer hydrogen bonds might reflect a more flexible or less stable interaction, potentially impacting the efficacy of these ligands in stabilising or modulating the protein function.

## 4. Conclusions and Future Perspective

Our in-silico analysis showed that asoprisnil exhibited the most favourable pharmacokinetic and pharmacodynamic properties, with no potential toxicity, compared to the four investigated ligands (including the reference ligand, mifepristone). Molecular dynamic simulation analysis also showed that asoprisnil had the most binding potential and stability with GR, with a binding energy of −62.35 kcal/mol, performing better than mifepristone, which had a binding energy of −57.08 kcal/mol. This makes asoprisnil a potential pharmacotherapeutic agent in stress-related mental disorders. Also, both mifepristone and asoprisnil can be further optimised (now that we know how they interact with GR) to be more effective for stress-associated psychiatric disorders.

Further studies, such as X-ray crystallography, nuclear magnetic resonance (NMR), surface plasma resonance, enzyme-linked immunosorbent assay (ELISA), and cell-based assays, are crucial to thoroughly evaluate their therapeutic potential, cytotoxicity, inhibitory properties, and molecular interactions. Further studies should also focus on the in vitro and in vivo validation of asoprisnil interaction; the efficacy, safety, and long-term impact on stress-related pathways (at the molecular and subcellular level); and clinical trials to evaluate its therapeutic potential in patients with stress-related mental disorders.

A limitation observed with asoprisnil in this study is its poor solubility and inability to permeate the blood–brain barrier. However, poor solubility is also observed with our reference ligand, mifepristone. One solution to overcome this challenge will be to use methods that improve the solubility of molecules, such as hot-melt extrusion, electrospinning, freeze-drying, and 3D printing or a combination of these [76]. These techniques enhance solubility by altering their physical form or formulation [77]. Hot-melt extrusion, for instance, creates amorphous solid dispersions that improve drug dissolution rates by increasing surface area and enhancing wettability [77]. Freeze-drying preserves molecular structure and reduces particle size, further aiding solubility [78]. Nanotechnology can also be used to optimise and modify these molecules to increase their permeability and cross the blood–brain barrier through mechanisms such as carrier-mediated transport (CMT), active transport and retention (ATR), and enhanced permeability and retention (ERP) using endocytosis and transcytosis pathways [79,80,81]. These methods are crucial in pharmaceutical development to overcome solubility challenges, thereby improving drug bioavailability and therapeutic efficacy.

## Figures and Tables

**Table 1:** Predicted pharmacokinetics (**A**) and toxicity (**B**) analysis of mifepristone, asoprisnil, campestanol, and stellasterol.

(A)					
	Pharmacokinetics				
	Molecular Weight g/mol	Water Solubility LogS (class)	Druglikeness (Lipinski’s)		Lipophilicity (Log P_o/w_)
**Mifepristone**	429.59	Poorly soluble	1 violation: MlogP > 4.15		4.60
**Asoprisnil**	449.58	Poorly soluble	0 violation		4.27
**Campestanol**	402.70	Moderately soluble	1 violation: MlogP > 4.15		6.77
**Stellasterol**	398.66	Moderately soluble	1 violation: MlogP > 4.15		6.58
(**B**)					
	**Predicted Toxicity Profile**				
		**Prediction (Probability)**			
**Classification**	**Target**	**Mifepristone**	**Asoprisnil**	**Campestanol**	**Stellasterol**
**Organ toxicity**	Hepatotoxicity	Inactive (0.96)	Inactive (0.73)	Inactive (0.79)	Inactive (0.75)
	Neurotoxicity	**Active** (0.78)	Inactive (0.85)	Inactive (0.56)	**Active** (0.57)
**Toxicity end points**	Carcinogenicity	Inactive (0.53)	Inactive (0.53)	Inactive (0.77)	Inactive (0.60)
	Immunotoxicity	**Active** (0.81)	**Active** (0.93)	**Active** (0.97)	**Active** (0.94)
	Mutagenicity	Inactive (0.90)	Inactive (0.57)	Inactive (0.87)	Inactive (0.96)
	Cytotoxicity	Inactive (0.80)	Inactive (0.75)	Inactive (0.88)	Inactive (0.96)
**Tox21-Stress response pathways**	Nuclear factor (erythroid-derived 2)-like 2/antioxidant responsive element (nrf2/ARE)	Inactive (0.97)	Inactive (0.88)	Inactive (0.93)	Inactive (0.73)
	Heat shock factor response element (HSE)	Inactive (0.97)	Inactive (0.88)	Inactive (0.93)	Inactive (0.73)
	Mitochondrial membrane potential (MMP)	**Active** (0.99)	Inactive (0.65)	Inactive (0.86)	Inactive (0.55)
	Phosphoprotein (tumor suppressor) p53	**Active** (1.0)	Inactive (0.81)	Inactive (0.99)	Inactive (0.99)
	ATPase family AAA domain-containing protein 5 (ATAD5)	Inactive (0.93)	Inactive (0.88)	Inactive (1.0)	Inactive (0.99)
**Cytochrome**	CYP1A2	Inactive (0.99)	Inactive (0.88)	Inactive (0.90)	Inactive (0.98)
	CYP2C19	**Active** (1.0)	Inactive (0.65)	Inactive (0.73)	Inactive (0.76)
	CYP2C9	Inactive (0.51)	Inactive (0.54)	**Active** (0.67)	**Active** (0.75)
	CYP2D6	**Active** (0.78)	Inactive (0.71)	Inactive (0.93)	Inactive (0.91)
	CYP3A4	**Active** (0.79)	Inactive (0.58)	Inactive (0.98)	Inactive (0.94)
	CYP2E1	Inactive (1.0)	Inactive (0.98)	Inactive (1.0)	Inactive (0.99)
**Acute inhalation toxicity**		**Yes**	No	No	No
**Acute oral toxicity**		**Yes**	No	**Yes**	No
**Acute dermal toxicity**		No	No	No	No
**Eye irritation and corrosion**		No	No	No	No
**Skin sensitisation**		No	No	No	No
**Skin irritation and corrosion**		No	No	**Yes**	No

**Table 2:** Ligand–protein docking result with GR.

Ligand	RMSD (Å)	Binding Affinity (kcal/mol)	Inhibition Constant (Ki)
Mifepristone	11.96	−12.39	821.48 pM
Asoprisnil	9.37	−11.68	2.76 nM
Campestanol	10.15	−8.90	300.27 nM
Stellasterol	11.90	−12.07	1.41 nM

## Data Availability

The data generated for this study are included within the article.

## Supplementary Materials

The following supporting information can be downloaded at https://www.mdpi.com/article/10.3390/biomedicines12122745/s1: Figure S1: BOILED-Egg model of ligands. BBB—blood–brain barrier; HIA—human intestinal absorption. The white region represents the physicochemical space where molecules have the highest likelihood of gastrointestinal absorption. The yellow region (yolk) represents the space where molecules are most likely to permeate the brain. These areas can overlap. Blue circles represent P-glycoprotein positive substrate (PGP+), and red circles represent P-glycoprotein negative substrate (PGP-).
