# Supplementary material for: Asoprisnil as a Novel Ligand Interacting with Stress-Associated Glucocorticoid Receptor
Source: Biomedicines. 2024 Nov 30;12(12):2745. doi: 10.3390/biomedicines12122745 (PMC11726916; doi:10.3390/biomedicines12122745)
Supplement: Supplementary file 1 [file biomedicines-12-02745-s001.zip › biomedicines-3318345-supplementary.pdf]

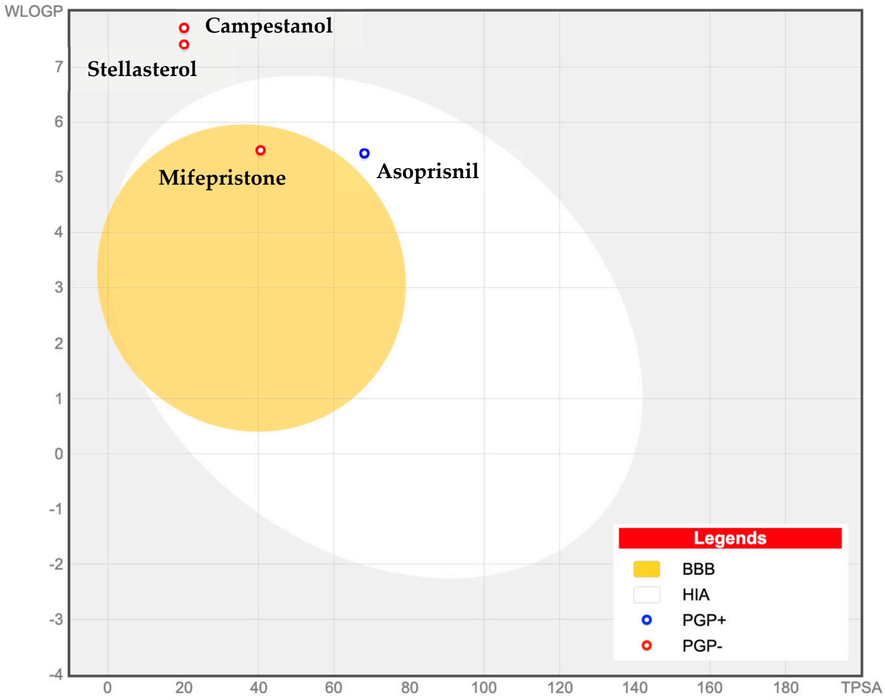

Figure S1. BOILED-Egg model of ligands. BBB - blood-brain barrier, HIA - human intestinal absorption. The white region represents the physicochemical space where molecules have the highest likelihood of gastrointestinal absorption. The yellow region (yolk) represents the space where molecules are most likely to permeate the brain. These areas can overlap. Blue circles represent P-glycoprotein positive substrate (PGP+), and red circles represent P-glycoprotein negative substrate (PGP-).
